# Fixed vs. variable light quality in vertical farming: Impacts on vegetative growth and nutritional quality of lettuce

**DOI:** 10.1371/journal.pone.0285180

**Published:** 2023-05-17

**Authors:** Yuyao Kong, Krishna Nemali

**Affiliations:** Department of Horticulture and Landscape Architecture, Purdue University, West Lafayette, Indiana, United States of America; University of Tsukuba, JAPAN

## Abstract

Lettuce (*Lactuca sativa*) is commonly produced in vertical farms. The levels of nutritionally important phytochemicals such as beta-carotene (precursor to vitamin A) are generally low in lettuce. In this study, we investigated the benefits of variable lighting strategy (i.e., varying the light quality during production) on maintaining plant growth and increasing the biosynthesis of beta-carotene and anthocyanin. We tested two variable lighting methods, using green and red romaine lettuce, namely (i) providing growth lighting (supports vegetative growth) initially (21 days) followed by a high percentage of blue light (supports biosynthesis of phytochemicals) at final stages (10 days) and (ii) providing a high percentage of blue light initially followed by growth lighting at final stages. Our results indicate that the variable lighting method with initial growth lighting and high percentage of blue at final stages can maintain vegetative growth and enhance phytochemicals such as beta-carotene in green romaine lettuce while both variable lighting methods were not effective in red romaine lettuce. In green romaine lettuce, we did not observe a significant reduction in shoot dry weight but there was an increase in beta-carotene (35.7%) in the variable compared to the fixed lighting method with growth lighting for the entire duration. The physiological bases for differences in vegetative growth and synthesis of beta-carotene and anthocyanin in the variable and fixed lighting methods are discussed.

## Introduction

About 70% of the world’s population is expected to live in urban areas by 2050 [1]. With population growth, it is critical that a continuous supply of fresh and nutritious food is available in urban areas. Unfortunately, moderate to high levels of hunger and malnutrition are already common in many urban areas of the world [2]. Alternate farming systems that are resilient to harsh climatic conditions and enable local food production are required in urban areas to increase the supply of fresh and nutritious food. Vertical farming, which involves growing food at multiple vertically stacked levels using controlled environmental conditions, is one of the methods to increase the availability of locally grown and fresh food in urban areas [3, 4]. In some regions experiencing extreme climatic conditions, vertical farming is the only method available for local food production. Moreover, low-cost and home-scale indoor vertical farms can enable fresh food production in low-income communities, thereby aiding in minimizing existing gaps in food equity.

At present, leafy greens are the major commodity produced in vertical farms [5] due to their fast growth rate and short cropping cycle. It can be challenging and economically may not be feasible to grow other crop species in vertical farming with the current level of technology. Albeit limited crop choices for production, vertical farming can still contribute significantly to the overall food availability and health of the urban population. Regular consumption of leafy greens, which are commonly produced in vertical farms, can aid in reducing the risk of chronic diseases in humans [6]. Among different leafy greens, lettuce is the most popular crop grown in vertical farms [7]. The productivity of crops such as lettuce grown in vertical farms (4.9 to 6.9 kg∙m^-2^ [8, 9]) is comparable to that of field-grown lettuce (3.6 to 4.1 kg∙m^-2^ [10]). Because crops can be grown at multiple levels per cycle and several cycles are possible per year, a significant quantity of lettuce can be grown in vertical farms to meet urban demand. However, the levels of nutritionally important phytochemicals such as beta-carotene (beta-car), a precursor to vitamin-A, are generally low in lettuce [9]. Vitamin-A deficiency is one of the major nutritional deficiencies globally [11, 12]. An enhancement of beta-car levels in lettuce not only ensures that the food produced is of higher nutritional quality but may also increase overall sales from vertical farming.

The increased biosynthesis of beta-car is associated with their photoprotective function in plants exposed to high-energy radiation. For example, a high percentage of blue light provided to plants enhanced the biosynthesis of beta-car in lettuce [9, 13]. Beta-car is integral to the photosystem II reaction core and physically quench triplet chlorophylls (^3^Chl) and singlet oxygen (^1^O_2_) from both the reaction center and the light-harvesting complex along with other pigments such as xanthophylls [14, 15]. Increased levels of beta-car are observed in plants exposed continuously to high energy radiation, to quench the excess ^3^Chl and ^1^O_2_. Further, the products of the reaction between ^3^Chl or ^1^O_2_ and beta-car aid as a signal for triplet stress by translocating into nucleus and eliciting subsequent genetic regulation for photoprotection and repair [15].

Manipulating light quality to enhance beta-car levels in lettuce appears to be intriguing and commercially feasible in vertical farms. Light emitting diode (LED)-based light fixtures with a ‘fixed’ light quality are commonly used in vertical farming [9]. These light fixtures provide a fixed percentage of different wavelengths (e.g., blue and red light) to plants during the entire growth period. Using these fixtures, plants can be provided with a light quality that either enhances vegetative growth or phytochemicals, but not both. For example, a high percentage of blue light (30 to 50%) can be provided as fixed lighting to increase the levels of beta-car. However, such a light recipe was also shown previously to reduce the vegetative growth of lettuce [16–18]. Plant responses such as decreased leaf expansion [19] and leaf angle [20], and increased percentage of shaded leaf area [21] were observed when exposed to a high percentage of blue light. A decrease in leaf area can reduce the overall biomass production in lettuce [22, 23] due to decreased light interception. On the other hand, a high percentage of low-energy radiation such as red light (70 to 90%) provided in fixed lighting can increase leaf expansion and shoot biomass in lettuce [24–26] but the levels of phytochemicals are reduced in lettuce [9, 13, 27]. Therefore, it can be challenging to increase both vegetative growth and the levels of nutritionally important phytochemicals using a ‘fixed’ light quality during growth.

In contrast to fixed lighting, the ‘variable’ lighting strategy involves changing light quality provided to plants by the growth stage. Compared to fixed lighting, the variable lighting strategy may allow for more control of plant growth and biosynthesis of phytochemicals during production [28]. As light quality effect on the synthesis of phytochemicals was reported to be more pronounced at mature growth stages [29], a variable light quality that enhances phytochemical biosynthesis may be provided at final stages. However, research-based information related to the effects of variable lighting on both vegetative growth and levels of nutritionally important phytochemicals in lettuce is limited. Such information is critical for manufacturers to develop variable LED light fixtures and for plant physiologists to optimize variable lighting recipes in vertical farms.

In this research, we tested two variable lighting methods, using green and red romaine lettuce, including (i) providing growth lighting (supports vegetative growth) initially (21 days) followed by a high percentage of blue light (supports biosynthesis of phytochemicals) at final stages (10 days) and (ii) providing a high percentage of blue light initially followed by growth lighting at final stages. The objectives of the study were to (1) quantify the effects of the two variable lighting methods on lettuce vegetative growth and levels of phytochemicals and (2) understand the physiological basis for observed differences in vegetative growth and phytochemical levels in plants subjected to the two variable lighting methods.

## Material and methods

### A. Plant material, growing system, and environmental conditions

We selected red and green lettuce varieties to test in this study as they exhibit contrasting differences in vegetative growth and phytochemical levels. Generally, green varieties show increased growth but the levels of phytochemicals are lower than that of red varieties [16, 17]. As romaine lettuce is among the popular groups, varieties belonging to green (cv. Amadeus) and red (cv. Intred) romaine lettuce were seeded in plug flats (72-cell; 3.5 cm × 3.5 cm × 5.9 cm, 30.2 mL per cell, Landmark Plastic, Akron, Ohio, USA) filled with a soilless substrate (80% peat, 15% perlite, and 5%vermiculite, BM2, Berger, Saint Modeste, Canada). The plug flats were placed under a mist system in a greenhouse to ensure uniform germination. After 10 days from sowing, all seedlings were transplanted into plastic pots (10.6 cm × 10.6 cm × 8.4 cm, 943 mL, Kord Products Ltd, Brampton, ON, Canada) filled with the same soilless substrate used for germination, and grown in a custom-built vertical farm.

The custom vertical farm was built using chrome-wire shelves [1.22 m (length) × 0.61 m (width) × 1.37 m (height), H-6948, Uline, Pleasant Prairie, WI, USA]. Two LED fixtures (0.6 m × 0.6 m, Applied Electronic Materials, Fort Wayne, USA) containing separate circuits for blue (450 ± 18 nm) and red (660 ± 19 nm) LEDs (Oslon SSL, Osram, Munich, Germany) were installed at the top of the chrome-wire shelves. Each light fixture comprised five light bars (60 cm long) each with six red and six blue LEDs. The individual LEDs were paced 10 cm apart in each circuit and emitted light at a 120° angle from the source. Red and blue light intensities in the light fixtures were adjusted using a controller (Time-Keeper MAX, Touch-Plate Light Controls, Fort Wayne, IN, USA). The custom vertical farm was located inside a glass greenhouse. To provide plants with sole-source LED lighting and prevent sunlight from reaching plants, the vertical farm was covered with two layers of black cloth (WeedBlock, Jobes Co., Waco, TX, USA). The cloth allowed air movement but reduced sunlight transmission into the vertical farm. Temperature and relative humidity were controlled by the greenhouse heating, cooling, and ventilation system. The CO_2_ concentration was not measured but deemed to be at the ambient level. Plants were watered as needed with a water-soluble fertilizer containing 20% N-4.4% P-16.6% K (20-10-20, Peters Professional, Summerville, SC, USA) at an electrical conductivity (EC) level of 1.7 ± 0.04 dS·m^-1^ and pH of 5.8 ± 0.04. The plants were grown for a period of 31 days in the vertical farm.

### B. Treatments

**Fig 1:**
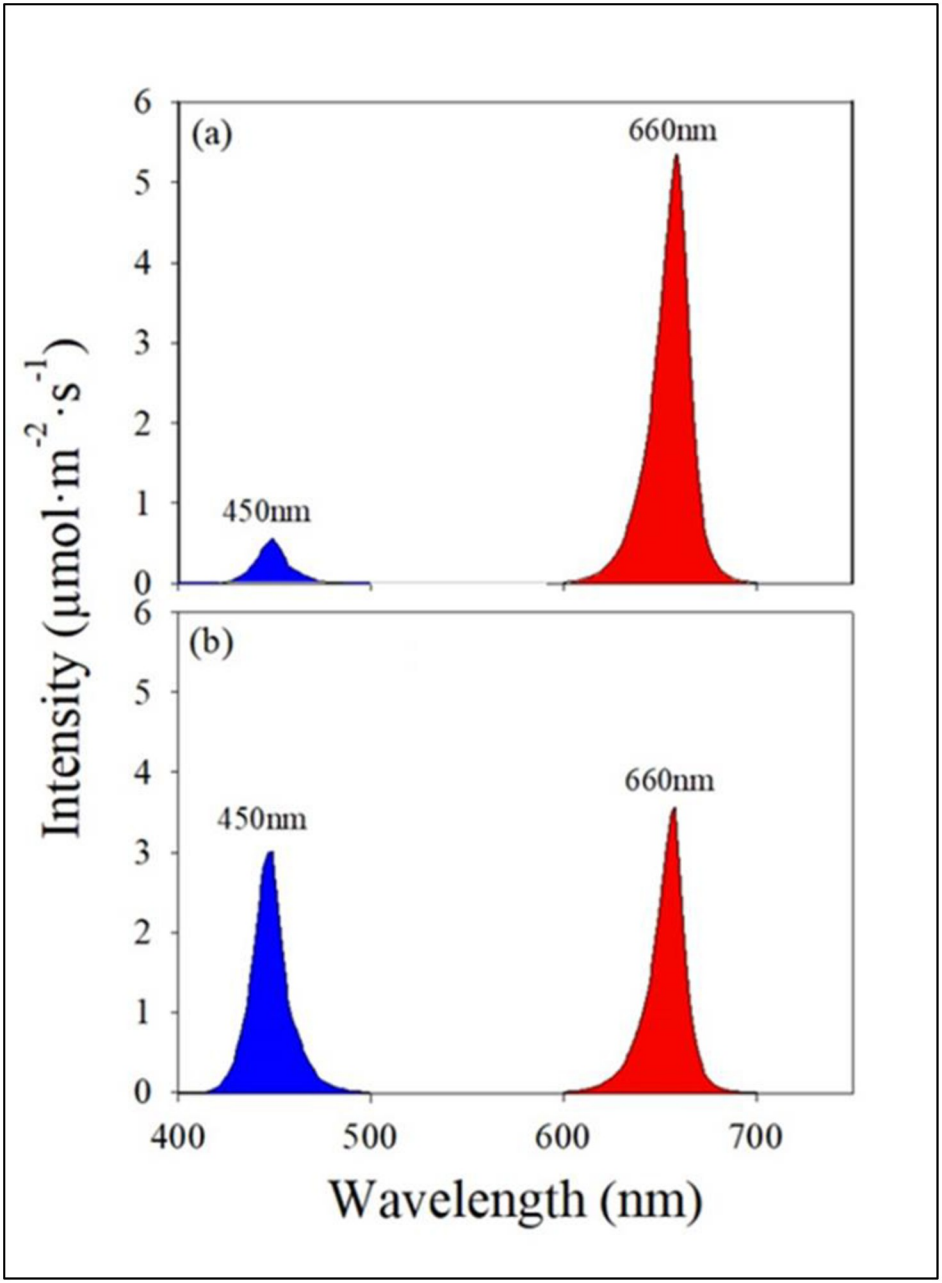
Spectral composition and peak wavelength of light in the two light quality treatments including (a). low percentage of blue (L) and (b). high percentage of blue (H) light. The variable light treatments were created by shifting plants from L to H and vice versa on the 21^st^ day of the experiment and exposing to the new treatment for 10 days.

The treatments included two light qualities, two varieties, and two lighting methods in the study. The light quality treatments included two levels of blue light in the total light including low (approximately 10%) and high (approximately 50%), with the remaining light provided as red light (Fig 1). The low and high blue light quality treatments were intended to increase vegetative growth and phytochemicals, respectively. The study included green and red leaf romaine varieties, which were selected based on inherent differences in growth and pigment levels. Plants were subjected to two lighting methods including ‘fixed’ and ‘variable’. The fixed method consisted of growing plants in the low [hereafter ‘L’] or high [hereafter ‘H’] blue light quality treatment for 31 days. The variable method consisted of growing plants in the L for 21 days followed by H for 10 days [hereafter ‘LH’] or growing plants in the H for 21 days followed by L for 10 days [hereafter ‘HL’]. The variable lighting method was achieved by shifting two plants of each variety from L to H or H to L treatment on the 21^st^ day of the study.

### C. Measurements

Photosynthetic photon flux density (*PPFD*), light quality, and air temperature were measured below each light fixture (i.e., corresponding to a light quality treatment). The air temperature was measured continuously using a thermistor (ST-100, Apogee Instruments, Logan, UT, USA) connected to a datalogger (CR 1000, Campbell Scientific, Logan, UT, USA) under each LED fixture and averaged for the growth period. The *PPFD* and light quality were measured using a spectroradiometer (SS-110, Apogee Instruments) at the start and end of the study from the same location. The measurements were made at four different locations under each LED fixture. Relative humidity measurements were recorded by the climate control system in the greenhouse (Priva, Camarillo, CA, USA). Average air temperature, daily light integral (DLI), and photoperiod inside the vertical farm were 22.0 ± 0.52 (day)/19.7 ± 0.30 (night) °C, 11.3 ± 0.06 mol·m^-2^·d^-1^, 24 h, and relative humidity of the greenhouse during the study was 62 ± 12.5%, respectively.

**Fig 2:**
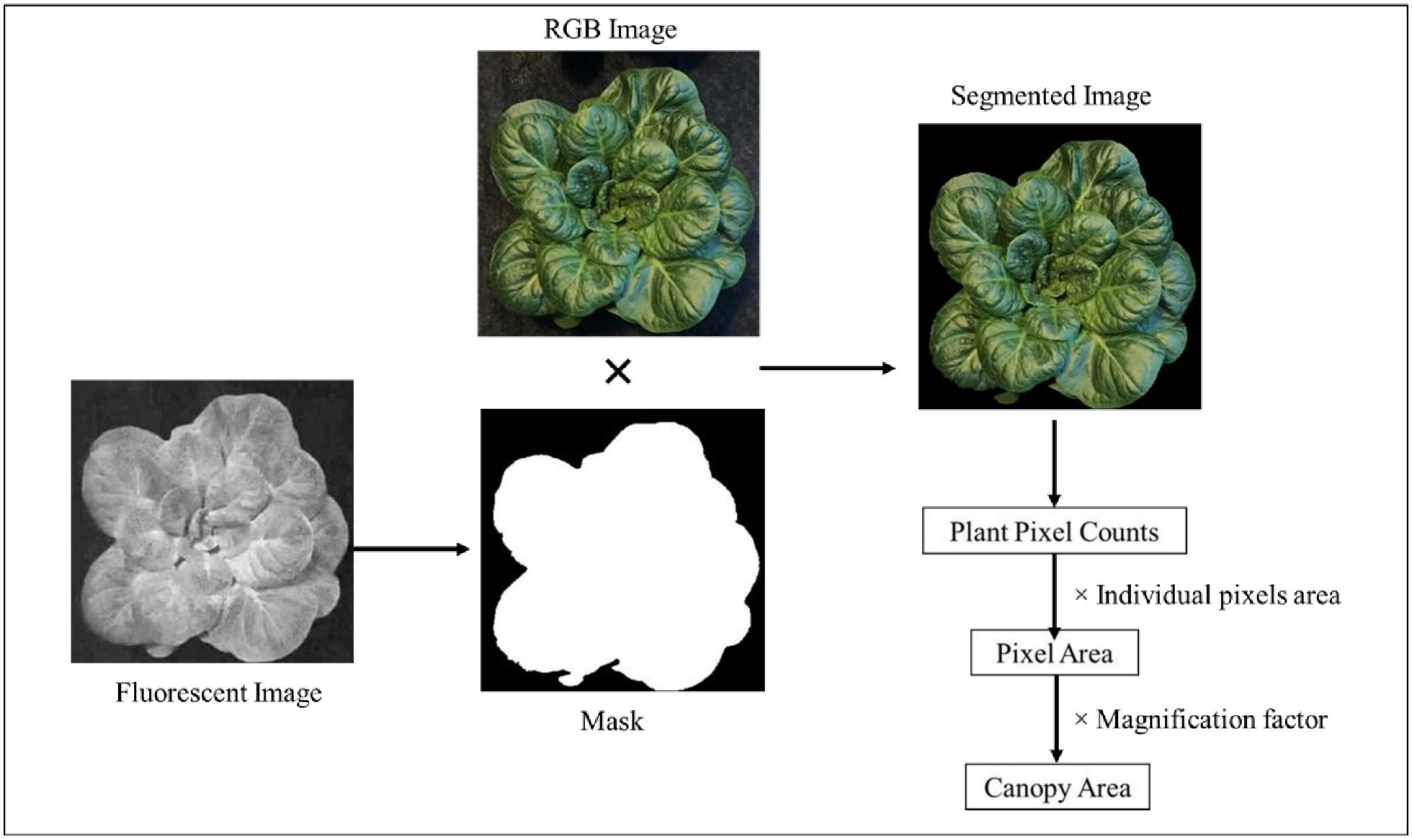
Canopy area estimation from plant images. The RGB and fluorescent images were captured by a camera, A mask was developed from the fluorescent image was used to remove background or segment plant in the RGB image. The plant pixel numbers are counted using the segmented image, pixel area was determined by multiplying total pixels with individual pixel area, and the canopy area was calculated by multiplying total pixel area with a magnification factor.

Non-destructive growth measurements included canopy area estimation on day 21 (CA_21_, cm^2^·plant^-1^). These measurements were carried out as described by Adhikari and Nemali [30] using an imaging system (TopView phenotyping system, Aris B.V. Eindhoven, the Netherlands). Briefly, RGB or color images plants were constructed in addition to a fluorescent image (Fig 2). A mask based on the fluorescence image was used for segmenting the plant areas from the background in the image. Image processing software automatically counted plant pixel numbers in the segmented image and calculated canopy area by multiplying total pixels with individual pixel area (0.001 mm^2^) and a magnification factor (100).

Plants were harvested on day 31 to measure shoot dry weight (SDW, g·plant^-1^) and total leaf area (LA, cm^2^·plant^-1^). Leaves belonging to a plant were separated and ran through the rollers of a leaf area meter (LI-3100C, Li-Cor Biosciences, Lincoln, NE, USA) to measure LA. The separated leaves and remaining plant material belonging to a plant were collected in a paper bag after LA measurement and the bag was placed in a forced-air oven maintained at 70°C for 5 days to measure SDW. As biomass is directly related to total carbon assimilation in photosynthesis, biomass per unit area (BMA, g∙100 cm^-2^) is proportional to growth period averaged carbon assimilation rate per unit leaf area in plants. It was calculated from SDW and LA as follows:

$$BMA=\frac{SDW\;\times 100}{LA}$$

The phytochemical levels were measured on a fresh weight basis at the harvest stage using a spectrophotometer (GENESYS 180 UV-Vis, Thermo Fisher Scientific, Waltham, MA, USA). Samples were collected from two mature leaves of each plant. The leaves were selected randomly from different levels on each plant. The levels of phytochemicals including beta-car (mg·100g^-1^) and anthocyanins (antho, ΔOD·g^-1^) were measured as described by Nagata and Yamashita [31] and Kong and Nemali [9], respectively. Leaf samples were stored in liquid nitrogen immediately after separating from plants. For beta-car, 0.2 g of the ground tissue was mixed with 1.8 ml acetone-hexene (2:3, v/v) solvent and stored overnight in the dark. The supernatant was 10-fold diluted and the absorption (A) at 663 nm (A_663_), 645 nm (A_645_), 505 nm (A_505_), and 453 nm (A_453_) was measured using the spectrophotometer. For antho, 0.1 g of the ground tissue was mixed in 4 ml of pre-cooled (4°C) 1% HCL-methanol solution (v/v) and stored in dark at 4°C for 20 minutes. The supernatant was then 10-fold diluted and the absorption was measured at 530 nm (OD_530_) and 600 nm (OD_600_) using the spectrophotometer. The concentration of beta-car was calculated as follows [31]:

$$\text{beta-car}\;\left(\text{mg}\cdot 100\;{\text{mL}}^{-1}\right)=0.216\cdot {\text{A}}_{663}-1.22\cdot {\text{A}}_{645}-0.304\cdot {\text{A}}_{505}+0.452\cdot {\text{A}}_{453},$$

This value was multiplied by extraction volume (1.8 mL) and dilution factor (10) and divided by sample weight (0.2 g) to convert beta-car level on a fresh weight basis (mg·100 g^-1^). The concentration of antho was calculated as follows [9]:

$$\text{antho}\;\left(\Delta \text{OD}\cdot {\text{g}}^{-1}\right)=\left({\text{OD}}_{530}-{\text{OD}}_{600}\right)$$

### D. Experimental design and statistical analyses

**Fig 3:**
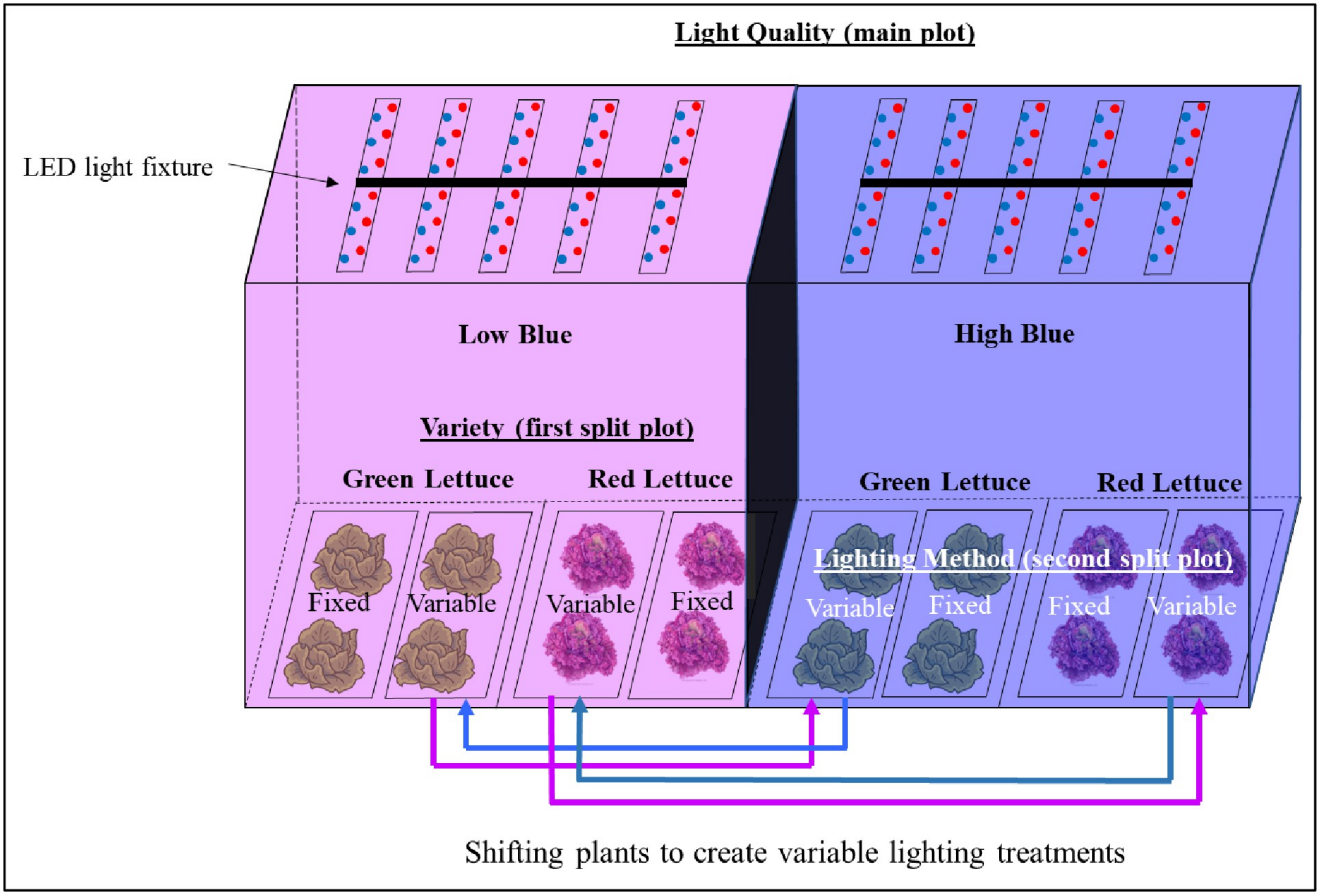
Experimental layout showing light fixtures and arrangement of plants in treatments associated with main, first split, and second split plots in a replication.

We conducted two similar experiments to increase the power of statistical comparison in the study. Both experiments were laid out in a split-split plot design with light quality as the main plot, variety as the first split, and lighting method as the second split (Fig 3). There were four and three replications of the main plot in the first and second experiments, respectively. In both experiments, an experimental unit consisted of two plants from a lighting method belonging to a variety within a light quality treatment. In total, there were 32 and 24 plants in the first and second experiments, respectively. The measurements of CA_21_ and antho were collected from the second experiment. The remaining measurements were collected from both experiments. A separate analysis for each variety had to be conducted to ensure normality and homoscedasticity of residuals. This reduced the design to a split-plot model for each variety with light quality as the main plot and lighting method as the split-plot. The data were analyzed using ‘GLIMMIX’ procedure of the statistical analysis software (SAS ver 9.4, Cary, NC) using experiment, replication, and replication × light quality as random variables. Least-square means for the main and interaction effects were separated using the Tukey-Kramer procedure. A pre-determined alpha value of 5% (*P*-value ≤ 0.05) was considered statistically significant for all statistical analyses.

## Results and discussion

### A. Differences in light quality

**Fig 4:**
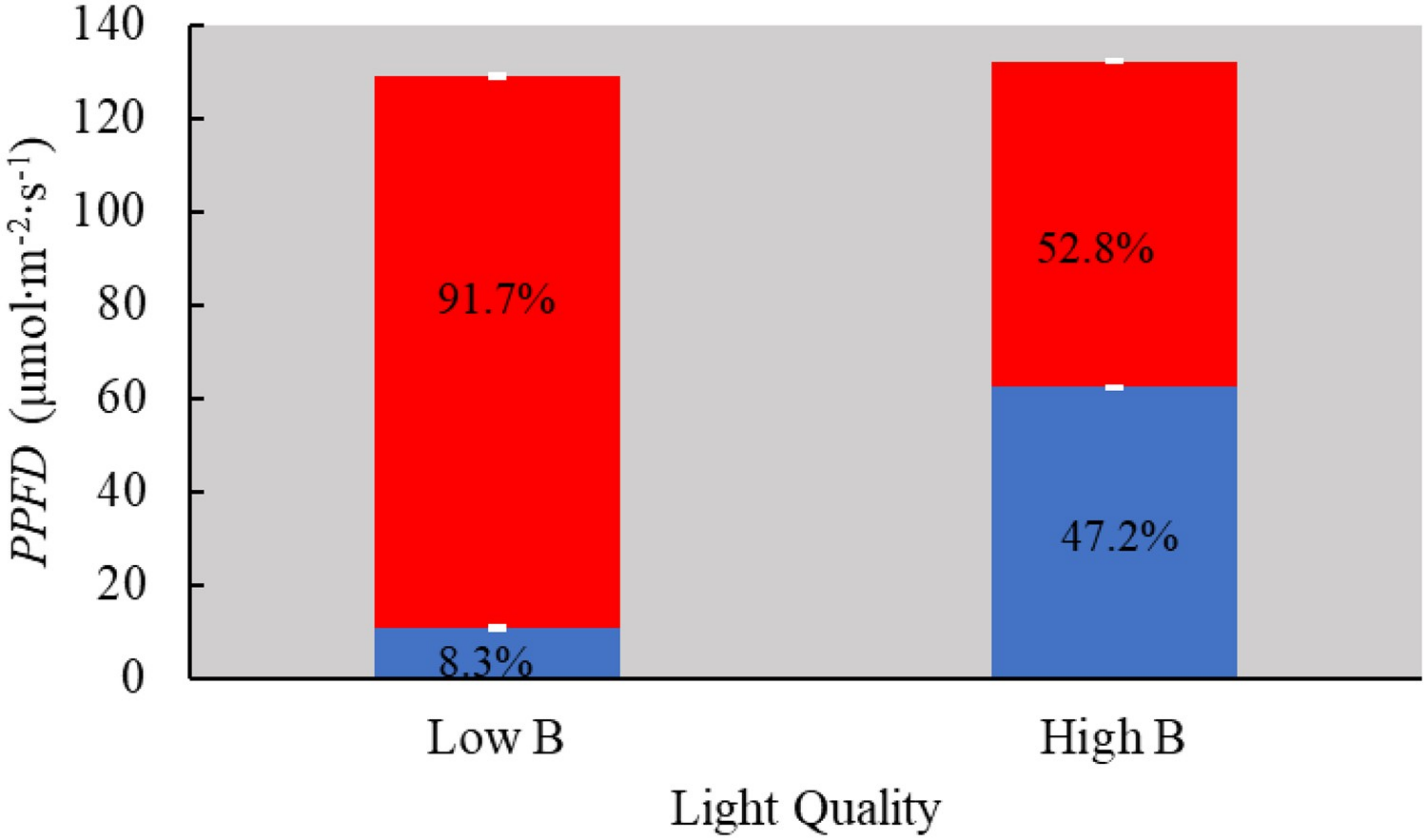
Th photosynthetic photon flux density (*PPFD*) of total, red, and blue light in the two light quality treatments. The measurements were collected using a spectroradiometer at four different locations. Total *PPFD* can be visualized by the size of the overall bar. Error bars represent the standard error of the mean of each light quality treatment. Numbers inside the bars indicate percentages of red and blue light in the total light.

There were no differences in *PPFD* between L and H treatments provided to plants in our study (Fig 4). However, the spectral composition of light received by plants was significantly different between the two light quality treatments (Figs 1 & 4). The blue light percentages were 8.3 and 47.2% in the L and H treatments, respectively. The red light percentages were 91.7 and 52.8% in the L and H treatments, respectively. The peak for blue and red wavebands was 450 and 660 nm with a full width of 25 nm at the half maximum rise (Fig 1).

The average DLI of 11.3 mol·m^-2^·d^-1^ provided to plants in our study was close to the recommended value of 12 to 14 mol·m^-2^·d^-1^ for lettuce [32]. The recommended percentage of red light in the total light for optimal lettuce growth vary between 65 to 90 [33–35] whereas phytochemical levels are usually enhanced when the percentage of blue light ranges between 30 and 50 [13, 24, 36]. Given this, plants in the L and H treatments were exposed to a light quality that enhances vegetative growth and biosynthesis of phytochemicals respectively during the entire growth period. On the other hand, the LH and HL treatments were exposed to a light quality that enhances phytochemical biosynthesis and vegetative growth respectively during the final growth stages.

### B. Green romaine lettuce

#### (i). Vegetative growth

The canopy area on day 21 was significantly affected by the light quality treatment in green romaine lettuce (Table 1). The average CA_21_ was higher in the L and LH than in the H and HL treatments. This is expected as low levels of blue radiation favor canopy expansion. Further, this indicates that canopy size was not different between L and LH or H and HL on day 21 i.e., before shifting plants.

**Table 1:** Effect of light quality (LQ) and lighting method (LM) on canopy area on day 21 (CA_21_), total leaf area (LA), biomass per unit area (BMA), and shoot dry weight (SDW) in green romaine lettuce. Least-square means with standard error of the model (in parenthesis) are shown in the table. Statistical significance of the main and interaction effects of the fitted model is shown below the table. The symbols ‘*’ and ‘**’, indicate *P* values ≤ 0.05 and 0.005, respectively, and ‘n.s.’ indicates no statistical significance. The least-square means with a different letter are statistically different (Tukey-Kramer procedure).

Light quality	Lighting Method	Treatment	CA_21_	LA	BMA	SDW
(LQ)	(LM)	Name	cm^2^∙plant^-1^	cm^2^∙plant^-1^	g∙100 cm^-2^	g∙plant^-1^
Low Blue	Fixed	L	331.5 (18.72) a	1139.3 (88.87) a	0.362 (0.0213) a	2.7 (0.46) a
	Variable	LH	281.4 (18.72) a	1038.0 (88.87) ab	0.358 (0.0213) a	2.2 (0.46) a
High Blue	Fixed	H	218.4 (18.72) b	971.1 (88.87) b	0.274 (0.0213) b	1.0 (0.46) b
	Variable	HL	257.4 (18.72) b	1070.1 (88.87) ab	0.332 (0.0213) a	2.2 (0.46) a
**Model Effects**						
LQ			*	n.s.	**	**
LM			n.s.	n.s.	*	n.s.
LQ × LM			n.s.	*	*	**

A significant interaction between light quality and lighting method was observed for the LA in green romaine lettuce (Table 1). As expected, the LA was higher in L than in H. The LA in both LH and HL treatments was intermediate to both L or H treatments. A decrease in the leaf area of lettuce exposed to high-energy blue radiation was previously reported [17, 18, 37]. Further, intermediate nature of LA in LH and HL indicate that LA decreased marginally in LH compared to L and increased marginally in HL compared to H. The marginal differences in LA observed between LH and L or HL and H partly agree with the observed differences in SDW between LH and L or HL and L (Table 1).

The interaction between light quality and lighting method was significant for BMA in green romaine lettuce (Table 1). Similar to the SDW, there were no differences in BMA between LH and L whereas a higher BMA was observed in HL than in H. As observed for SDW, the BMA of green romaine lettuce was higher in the L than in the H. Further, the BMA of green romaine lettuce was not different between HL and L or LH treatments, similar to SDW responses. These results suggest that shifting plants from L to H treatment had no effect whereas shifting plants from H to L treatment had a significant positive effect on BMA. Overall, these results indicate a relatively stronger correlation between SDW and BMA in green romaine lettuce.

**Fig 5:**
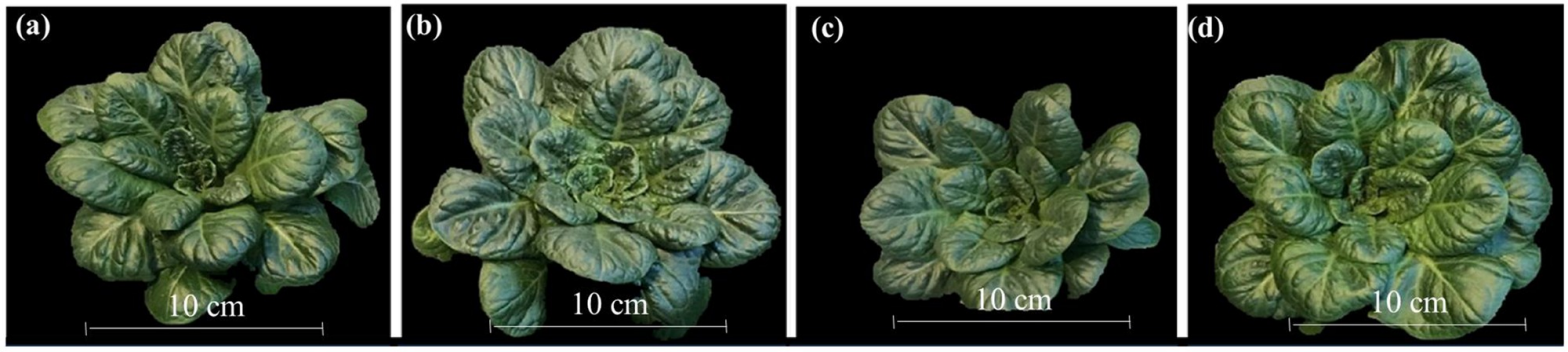
Representative green romaine lettuce plants from different light treatments. (a). low blue or L, (b). low to high blue or LH, (c). high blue or H, and (d). high to low blue or HL.

The interaction between light quality and lighting method was significant for SDW in green romaine lettuce (Table 1, Fig 5). This indicates that the effect of light quality on SDW depended on the lighting method. The SDW of green romaine lettuce was not different between LH and L, whereas it was higher in HL than in H. As expected, the SDW of green romaine lettuce was higher in the L than in the H, when the light quality was fixed. Further, the SDW of green romaine lettuce in HL was not different from either LH or L (Table 1, Fig 5). These results indicate that vegetative growth was more favored in the L than in the H treatment. Previous work by Son and Oh [16], and Lee et al. [25] supports our results that a high percentage of red in the total light, as in the L treatment, promotes vegetative growth. However, shifting plants from L to H during the final growth stages did not negatively affect SDW. Whereas, shifting plants from H to L during the final growth stages had a positive effect on SDW to the extent that the vegetative growth in HL was comparable to that of L and LH.

The observed differences in SDW can be collectively explained by the LA and BMA responses. The measurements of LA can be related to light absorption [38]. The BMA in lettuce is similar to leaf mass area measurements (as most of the shoot is comprised of leaves). Similar to leaf mass area, BMA is closely associated with the photosynthesis rate [39, 40] in plants. Therefore, LA and BMA can affect vegetative growth in plants. A marginal decrease in LA with no change in BMA between LH and L likely resulted in no significant differences in SDW. Likewise, a marginal increase in LA and a significant increase in BMA likely resulted in a significant increase in SDW in HL compared to H. A decrease in leaf expansion and vegetative growth was previously observed when plants were exposed to a high percentage of blue light [16–18].

#### (ii). Beta-carotene and anthocyanins

A significant interaction between light quality and lighting method was observed for beta-car levels in green romaine lettuce. The levels were significantly higher in LH than in L treatment, a 35.7% increase (Table 2). However, beta-car levels were lower in the HL than in the H treatment. Surprisingly, there were no differences in beta-car between the L and H treatments. Further, a higher beta-car level was observed in the LH than in the HL treatment. These results suggest that shifting plants from L to H during the final growth stage had a positive effect on the levels of beta-car, and shifting plants from H to L had a negative effect on beta-car levels.

**Table 2:** Effect of light quality (LQ) and lighting method (LM) on the levels of beta-carotene (beta-car) and anthocyanin (antho) in green romaine lettuce. Least-square means with standard error of the model (in parenthesis) are shown in the table. Statistical significance of main and interaction effects of the fitted model is shown below the table. The symbols ‘*’, ‘**’, and ‘***’ indicate *P* values ≤ 0.05, 0.005, and 0.0005 respectively, and ‘n.s.’ indicate no statistical significance. The least-square means with a different letter are statistically different (Tukey-Kramer procedure).

Light quality	Lighting Method	Treatment	beta-car	antho
(LQ)	(LM)	Name	mg∙100 g^-1^	ΔOD∙g^-1^
Low Blue	Fixed	L	4.08 (0.535) b	0.000 (0.0019) b
	Variable	LH	5.54 (0.535) a	0.002 (0.0019) b
High Blue	Fixed	H	4.89 (0.535) ab	0.018 (0.0019) a
	Variable	HL	3.74 (0.535) b	0.014 (0.0019) a
**Model Effects**				
LQ			n.s.	**
LM			n.s.	n.s.
LQ × LM			***	*

The interaction between light quality and lighting method was also significant for antho in green romaine lettuce (Table 2). The levels of antho were not different between the L and LH treatments but higher in the H than in the HL treatment. The antho levels were lower in the L than in the H. Compared to H and HL, the antho levels were several folds lower in L and LH treatments (Table 2). These results suggest that exposure to either H or L light quality during the final growth stage had a minimal impact on the biosynthesis of antho in green romaine lettuce.

Interestingly, biosynthesis of phytochemicals appears to be related to the duration of exposure to high levels of blue radiation in green romaine lettuce. The levels of beta-car increased in LH (after a relatively shorter exposure of 10 days) whereas the levels of antho were higher in H and HL treatments (after a prolonged exposure of 20 to 30 days). Although both beta-car and antho are involved in photoprotection, their biosynthesis appears to be tightly regulated such that there is no wastage due to redundancy. Moreover, an increase in the levels of beta-car observed in LH suggests that the initial acclimation responses related to photoprotection appear to happen in the chloroplast. Subsequent to this acclimation, other photoprotective mechanisms including biosynthesis of antho are likely triggered, based on several folds increase in antho levels observed in H and HL treatments. A recent study suggests that beta-car not only provides photoprotection to high light stress but also serves as a signal molecule to trigger genetic regulation for the subsequent photoprotection and repair in plants [15]. Although genetic regulation of beta-car and antho in plants is well understood, very little is known whether biosynthesis of beta-car either directly or indirectly regulates the levels of antho in plants. In this regard, it is interesting to note that a leucine-zipper transcription factor, Long Hypocotyl 5 (HY5), activates carotenoid and chlorophyll biosynthesis genes [41] in addition to activating anthocyanin structural genes [42]. This may suggest the presence of unknow interrelations between beta-car and antho biosynthesis in plants.

#### (iii). Physiological bases for growth differences

The observed marginal decrease and a significant increase in the vegetative growth in LH (compared to L) and HL (compared to H) treatments respectively was likely influenced by photosynthate partitioning during the final stages of growth. Leaf area in lettuce can increase by 18 to 22% of the existing area during the log growth phase [43], which corresponds to the final growth stages in our study. The exponential increase in leaf area can result in a rapid increase in photosynthate production during the log growth phase. It is likely that both vegetative growth and phytochemical biosynthesis processes competed for the photosynthate in LH treatment. However, relatively more photosynthates were likely partitioned for the biosynthesis of phytochemicals than vegetative growth in the LH. One likely reason for this preferential partitioning is to rapidly increase photoprotective mechanisms under high-energy radiation stress. On the other hand, the partitioning pattern was likely different in HL treatment during the final stages of growth. Our data shows a significant increase in BMA (carbon assimilation) in HL than in H treatment. This likely increased photosynthate production but partitioned them preferentially towards vegetative growth as there was no requirement for photoprotection. Phytochemicals such as antho can be readily degraded to support vegetative growth in plants [44]. It is likely that rapid vegetative growth in HL during the final growth stages was further supported by antho degradation, as evidenced by a decrease in their concentration in HL compared to H.

#### (iv). Model explaining the effects of variable lighting

**Fig 6:**
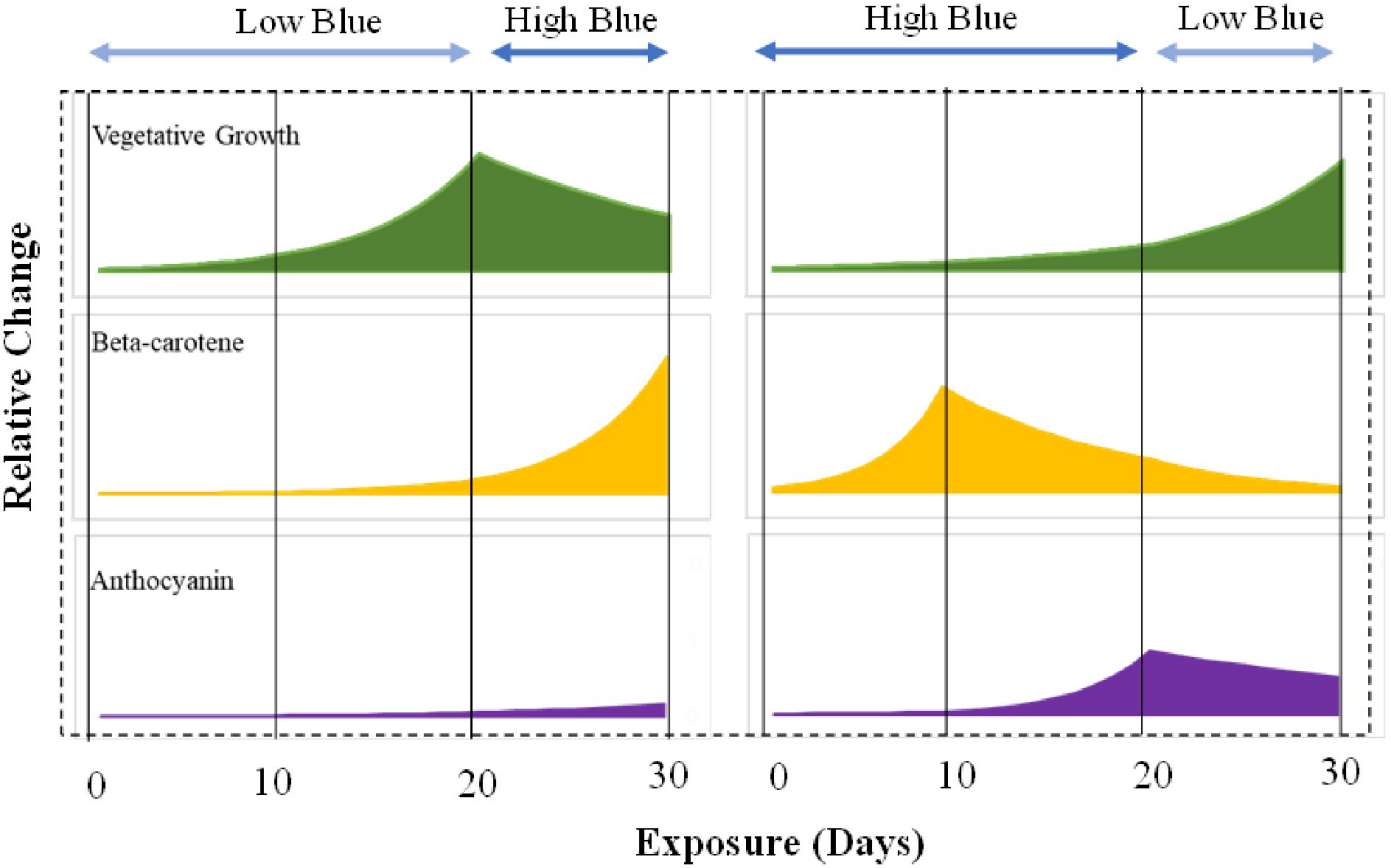
Proposed model for changes in vegetative growth, beta carotene, and anthocyanin in green romaine lettuce exposed to the two variable lighting treatments. The left panel shows the variable lighting method with a low percentage of blue radiation initially followed by a high percentage of blue radiation during the final growth stage and *vice versa* for the right panel.

Based on the results from the study, the following model on the effects of variable lighting is proposed (Fig 6). The Vegetative growth increased during the initial 21 days in the LH treatment due to the low percentage of blue in the total radiation (Fig 6). During this period, the requirement for photoprotection was minimal and likely resulted in a small increase in the levels of phytochemicals. When plants in the LH treatment were shifted to high blue treatment during the final growth stages, beta-car synthesis likely increased as a first response to photoprotection. The antho levels likely increase only after prolonged exposure to a high percentage of blue light, therefore their levels were likely unchanged in the LH treatment during the 10 days of exposure to high-energy radiation. A marginal decline in vegetative growth was observed in LH partly due to the partitioning of photosynthates preferentially for beta-car synthesis during the final 10-day exposure to high-energy radiation. On the other hand, the vegetative growth was slow in the HL treatment during the initial 21 days of exposure to a high percentage of blue light. During this time, beta-car levels likely increased initially to provide photoprotection whereas an increase in antho was observed likely later due to prolonged exposure to high-energy radiation. It is possible that beta-car degradation happened after prolonged exposure to support the biosynthesis of antho, especially when vegetative growth was slow. When plants were shifted from H to L during the final growth stages, a rapid increase in vegetative growth was observed. This rapid increase in vegetative growth was likely supported by an increase in BMA and the degradation of beta-car and antho. The degradation of phytochemicals likely leads to a decline in their concentrations.

### C. Red romaine lettuce

#### (i). Vegetative growth and phytochemicals

**Fig 7:**
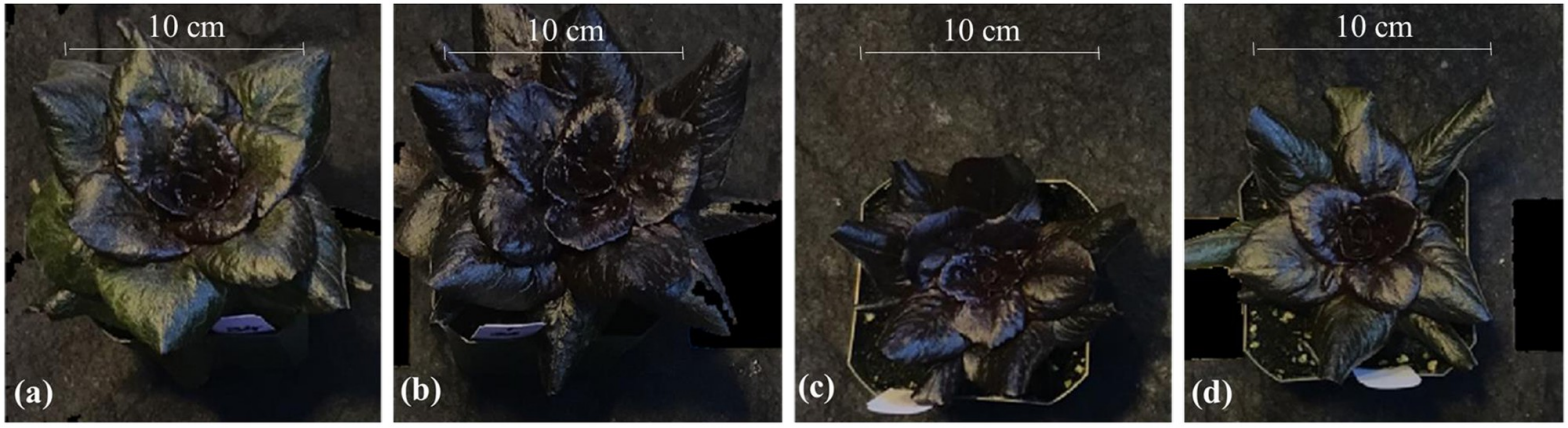
Representative red romaine lettuce plants from different light treatments. (a). low blue or L, (b). low to high blue or LH, (c). high blue or H, and (d). high to low blue or HL.

The effects of light quality were evident prior to shifting red romaine plants on day 21 as seen in higher CA_21_ in L and LH than in H and HL treatments (Table 3, Fig 7). These effects continued to the harvest stage when a significant main effect of light quality was observed on LA and BMA of red romaine lettuce, with higher values in the L and LH than in H and HL treatments (Table 3). The main effect of light quality was also significant on the SDW of red romaine lettuce (Table 3). The average SDW was higher in the L and LH than in the H and HL treatments. This suggests that a low percentage of blue light favors vegetative growth in red romaine lettuce. In addition, the higher SDW in L and LH than in H and HL treatments was due to higher CA_21_ leading to higher LA and higher BMA in red romaine lettuce. We did not observe any differences in beta-car or antho among the treatments in red romaine lettuce (Table 4).

**Table 3:** Effect of light quality (LQ) and lighting method (LM) on canopy area on day 21 (CA_21_), total leaf area (LA), biomass per unit area (BMA), and shoot dry weight (SDW) in red romaine lettuce. Least-square means with standard error of the model (in parenthesis) are shown in the table. Statistical significance of the main and interaction effects of the fitted model is shown below the table. The symbol ‘*’ and ‘n.s.’ indicate *P* values ≤ 0.05 and no statistical significance, respectively. In addition, *P* values close to statistical significance are shown. The least-square means with a different letter are statistically different (Tukey-Kramer procedure).

Light quality	Lighting Method	Treatment	CA_21_	LA	BMA	SDW
(LQ)	(LM)	Name	cm^2^∙plant^-1^	cm^2^∙plant^-1^	g∙100 cm^-2^	g∙plant^-1^
Low Blue	Fixed	L	167.3 (13.8) a	506.7 (97.64) a	0.213 (0.0317) a	1.1 (0.34) a
	Variable	LH	147.1 (13.8) a	500.3 (97.64) a	0.153 (0.0317) a	0.6 (0.34) a
High Blue	Fixed	H	95.6 (13.8) b	320.0 (97.64) b	0.136 (0.0317) b	0.3 (0.34) b
	Variable	HL	112.2 (13.8) b	303.2 (97.64) b	0.145 (0.0317) b	0.5 (0.34) b
**Model Effects**						
LQ			*	*	*	*
LM			n.s	n.s.	n.s.	n.s.
LQ × LM			n.s.	n.s.	0.06	n.s.

**Table 4:** Effect of light quality (LQ) and lighting method (LM) on the levels of beta-carotene (beta-car) and anthocyanin (antho) in red romaine lettuce. Least-square means with standard error of the model (in parenthesis) are shown in the table. Statistical significance of the main and interaction effects of the fitted model is shown below the table. The symbol ‘n.s.’ indicates no statistical significance. The least-square means with a same letter are not statistically different based on the Tukey-Kramer procedure.

Light quality	Lighting Method	Treatment	beta-car	antho
(LQ)	(LM)	Name	mg∙100 g^-1^	ΔOD∙g^-1^
Low Blue	Fixed	L	2.39 (0.276) a	0.94 (0.126) a
	Variable	LH	3.01 (0.276) a	1.04 (0.126) a
High Blue	Fixed	H	2.92 (0.276) a	1.27 (0.126) a
	Variable	HL	2.79 (0.276) a	1.15 (0.126) a
**Model Effects**				
LQ			n.s.	n.s.
LM			n.s.	n.s.
LQ × LM			n.s.	n.s.

The main effect of light quality on SDW, LA, and BMA of red romaine lettuce may be related to the levels of antho in red romaine lettuce plants. One of the functional roles of antho is to provide photoprotection by absorbing excess energy before it reaches chloroplasts [45–48]. Anthocyanins have a peak absorption at around 530 nm of the light spectrum and the range of absorbance can extend into shorter (blue) wavelengths [49]. In other words, anthocyanins absorb very little in the red waveband. When red romaine lettuce was provided with a low percentage of blue radiation, a relatively small proportion of the total light in the blue waveband was likely absorbed by antho in the leaves leaving most of the photons for chlorophyll absorption. On the other hand, when red romaine lettuce was provided with a high percentage of blue radiation, a relatively larger proportion of the total light in the blue waveband was likely absorbed by antho, leaving a relatively smaller number of photons for chlorophyll absorption. The difference in the available light for chlorophyll absorption may be the likely reason for relatively increased SDW, LA, and BMA of red romaine lettuce in the L and LH than in H and HL. In support, Tattini et al. [50] found that red basil leaves with enriched levels of antho showed ‘shade syndrome’ when grown in full sunlight, indicating that fewer photons arrived at the chloroplast due to the screening effect of antho.

Biosynthesis of phytochemicals in lettuce is favored to provide photoprotection from high-energy radiation. No effect of light quality on the levels of phytochemicals in red romaine lettuce is likely due to (a) already existing high-level of photoprotection from inherently high levels of antho in the leaves and (b) relatively reduced intensity of blue photons reaching chloroplast and reduced levels of high-energy radiation stress due to the screening of blue radiation by antho in the cells.

### D. Qualitative comparison between green and red romaine lettuce

The levels of antho were qualitatively much higher, beta-car lower, and plant size smaller in red than in green romaine lettuce (Figs 5 and 7; Tables 1 and 3). Previously other studies reported slower growth of red leaf compared to green leaf lettuce cultivars [17, 51]. The observed differences in vegetative growth between green and red varieties may be related to inherently high levels of antho in red romaine lettuce. Anthocyanins are synthesized in the mesophyll cells that are closer to the epidermis and sequestered in the vacuole [52, 53]. Biosynthesis of antho can have a relatively large metabolic cost on plants [54, 55]. The process involves the synthesis of several enzymes and the transportation of antho molecules into the vacuole. Further, antho biosynthesis competes with the growth for monosaccharides produced in photosynthesis [56, 57]. Collectively, the biosynthesis of antho can have tradeoffs with the vegetative growth. Based on our results, we hypothesize that inherent increases in antho also likely reduced the levels of beta-car to minimize the redundancy-related wastage of resources for photoprotection.

### E. Recommendations on variable lighting

In green romaine lettuce, the SDW was not statistically different and the beta-car level increased by 35.7% in the LH compared to the L treatment (Tables 1 and 2). Although SDW increased, the beta-car level decreased by 23.5% in the HL compared to the H treatment. Between the two variable lighting methods, a decrease of 48.1% in the beta-car level was observed in the HL than in the LH treatment. Based on these results, the LH is superior to the HL treatment as vegetative growth can be maintained while beta-car was enhanced. Therefore, variable lighting that promotes vegetative growth during the initial stages of growth followed by a light quality that enhances phytochemical biosynthesis during the final stages of growth is recommended to enhance the levels of beta-car with a minimal negative impact on plant growth in green romaine lettuce. The absence of either the main or interaction effect associated with the lighting method suggests that the red leaf variety may not be sensitive to variable lighting.

## Conclusions

The objectives of our study were to quantify the effects and understand the physiological basis for observed plant responses in the two variable lighting methods on vegetative growth and levels of phytochemicals such as beta-car and antho in green and red romaine lettuce. Our research indicates that it is possible to maintain vegetative growth and enhance phytochemicals such as beta-car in green romaine lettuce using a variable lighting strategy that enhances vegetative growth during initial growth and biosynthesis of phytochemicals for a short period (7 to 10 days) during the final stages of growth. However, the optimal time for variable lighting that results in maximum benefit may need further investigation. In addition, we found that red romaine lettuce, with inherently high levels of antho, may not respond to variable lighting methods. Our results indicate that variable lighting may not increase antho levels in green romaine lettuce, compared to the inherently high levels observed in red romaine lettuce. However, red romaine lettuce with high levels of antho can be mixed with green romaine lettuce treatment to provide nutritionally rich lettuce to consumers. Although we provided variable lighting by manually shifting plants between L and H treatments, it is possible to automate the process with improvements in engineering technology. The developed technology may need to be optimized in the future to ensure that variable lighting is economically feasible in vertical farming.

## Supporting information

S1 Data (XLSX)
